# Low Density Lipoprotein-Receptor Related Protein 1 Is Differentially Expressed by Neuronal and Glial Populations in the Developing and Mature Mouse Central Nervous System

**DOI:** 10.1371/journal.pone.0155878

**Published:** 2016-06-09

**Authors:** Loic Auderset, Carlie L. Cullen, Kaylene M. Young

**Affiliations:** Menzies Institute for Medical Research, University of Tasmania, Hobart, Tasmania, 7000, Australia; University of Queensland, AUSTRALIA

## Abstract

The low density lipoprotein-receptor related protein 1 (LRP1) is a large endocytic cell surface receptor that is known to interact with a variety of ligands, intracellular adaptor proteins and other cell surface receptors to regulate cellular behaviours ranging from proliferation to cell fate specification, migration, axon guidance, and lipid metabolism. A number of studies have demonstrated that LRP1 is expressed in the brain, yet it is unclear which central nervous system cell types express LRP1 during development and in adulthood. Herein we undertake a detailed study of LRP1 expression within the mouse brain and spinal cord, examining a number of developmental stages ranging from embryonic day 13.5 to postnatal day 60. We report that LRP1 expression in the brain peaks during postnatal development. On a cellular level, LRP1 is expressed by radial glia, neuroblasts, microglia, oligodendrocyte progenitor cells (OPCs), astrocytes and neurons, with the exception of parvalbumin^+^ interneurons in the cortex. Most cell populations exhibit stable expression of LRP1 throughout development; however, the proportion of OPCs that express LRP1 increases significantly from ~69% at E15.5 to ~99% in adulthood. We also report that LRP1 expression is rapidly lost as OPCs differentiate, and is absent from all oligodendrocytes, including newborn oligodendrocytes. While LRP1 function has been primarily examined in mature neurons, these expression data suggest it plays a more critical role in glial cell regulation–where expression levels are much higher.

## Introduction

Low density lipoprotein receptor related protein 1 (LRP1) is one of the largest members of the low-density lipoprotein receptor family, and binds a large variety of ligands to influence a range of cellular behaviors (reviewed [1]). While LRP1 is best known for its ability to mediate endocytosis [2,3], it can also operate as a co-receptor [4], or recruit non-receptor tyrosine kinases to its intracellular domain to mediate intracellular signal transduction [5]. Furthermore LRP1 can undergo proteolytic cleavage, reminiscent of notch or the amyloid precursor protein cleavage, to generate a soluble extracellular fragment [6,7] or a free intracellular domain, which has been shown to enter the nucleus and influence gene transcription [8–10]. Utilizing these signaling mechanisms LRP1 performs unique tissue-specific functions (reviewed [11]), and it is highly likely to mediate cell-type specific functions in the central nervous system (CNS).

LRP1 is widely expressed throughout the CNS. The majority of research examining LRP1 function in the brain has focused on its role in regulating amyloid precursor protein trafficking [12,13], amyloid β clearance from the brain parenchyma (reviewed [14]), and blood brain barrier permeability [15]. However LRP1 is also detected in mature neurons, particularly those of the entorhinal cortex, hippocampus [16] and cerebellum [17], and is critical for neuronal function. The selective deletion of *Lrp1* from differentiated neurons during mouse development, leads to behavioural and motor defects including hyperactivity, tremor and dystonia [18]. These effects are primarily due to the importance of LRP1 for regulating synaptic function, specifically at the post-synaptic density where it is thought to regulate the turnover and recycling of synaptic proteins [18,19]. More recently LRP1 was also shown to mediate the chemo-attraction and -repulsion of sensory neuron growth cones *in vitro* [20], and there is some evidence that LRP1 is expressed by astrocytes [21], microglia [22] and oligodendrocytes [23] *in vitro*, and by a sub population of radial glia in the embryonic mouse brain [24].

Studies characterising the expression of LRP1 in the CNS have often focused on a single stage of development, and examined gross regional expression or a single cell type, often *in vitro* (reviewed [1]). Therefore, when reading the literature, it is unclear which cells within the CNS actually express this receptor, and which do not. Recent microarray and RNA sequencing data have shown that *Lrp1* mRNA is highly expressed by neurons, astrocytes and microglia, as well as oligodendrocyte progenitor cells (OPCs) and newly formed oligodendrocytes in the early postnatal brain, but indicate that it is down-regulated as the cells differentiate into mature myelinating oligodendrocytes [25,26]. This is the first indication that LRP1 may be expressed by oligodendrocyte lineage cells in the healthy nervous system, but has not been verified at the protein level. Herein we characterise LRP1 expression within the developing and mature mouse brain and spinal cord. We report that LRP1 is expressed extensively throughout the CNS, being expressed at high levels by radial glia, neuroblasts, neurons, microglia, astrocytes and OPCs. However LRP1 was not expressed by mature oligodendrocytes in the brain or spinal cord, and was not expressed by parvalbumin-positive cortical interneurons–indicating that LRP1 is not generically expressed by all neural cell types.

## Experimental Procedures

### Animal Housing

All mice were purchased from Jackson Laboratories and maintained on a C57bl6 background and housed in Optimice micro-isolator cages (Animal Care Systems, Colorado, USA) on a 12 hour light/dark cycle at 20°C with uninhibited access to food and water. *Pdgfrα-CreER*^*T2*^ [27] mice were crossed with Cre-sensitive *Rosa26-YFP* [28] reporter mice. Both male and female mice were weaned at P35 and used in adulthood for experiments approved by the University of Tasmania Animal Ethics Committee.

### Genotyping

Ear biopsies were digested overnight in DNA extraction buffer (100mM Tris-HCl, 5mM EDTA, 200mM NaCl, 0.2% SDS and 120ng of proteinase k) at 55°C. Genomic DNA was then extracted by first precipitating cellular and histone proteins by cold incubation in 6M Ammonium Acetate (Sigma; A1542), followed by DNA with room temperature isopropyl alcohol (Sigma; I9516). The DNA pellet was washed in 70% Ethanol (Sigma; E7023), resuspended in autoclaved MilliQ water and used as template DNA to genotype the mice by polymerase chain reaction (PCR). The PCR was performed as a 25μL reaction containing 50–100ng DNA, 0.5μL of each primer (100nmol/mL; GeneWorks) and 12.5 μL GoTaq green master mix (Promega) in MilliQ water. To genotype mice expressing the *Rosa26-YFP* transgene we used three primers: Rosa26 wildtype forward AAAGTC GCTCT GAGTT GTTAT, Rosa26 wildtype reverse GGAGC GGGAG AAATG GATATG and Rosa26 YFP forward GCGAA GAGTTT GTCCT CAACC in a program of: 94°C for 4’, and 37 cycles of 94°C for 30”, 60°C for 45”, and 72°C for 60”, followed by 72°C for 10 minutes. To genotype mice expressing the *Pdgfrα-CreER*^*T2*^ transgene we used two primers: Cre5’ CAGGT CTCAG GAGCT ATGTC CAATT TACTG ACCGTA and Cre3’ GGTGT TATAA GCAAT CCCCA GAA in a program of: 94°C for 4’, and 34 cycles of 94°C for 30”, 62°C for 45”, and 72°C for 1’, followed by 72°C for 10’. The DNA was then separated by gel electrophoresis (1% w/v agarose in TAE containing SYBR-safe, ThermoFisher), and imaged using an Image Station 4000M PRO gel system running Carestream software.

### Western Blot

E13.5, P5 and P62 mouse brain protein lysates were produced by lysing tissue in RIPA cell lysis buffer (50mM Tris-HCL, 150mM NaCl, 1%NP-40, 1% Sodium Deoxycholate, 0.1% SDS and one phosphatase inhibitor tablet). Samples were centrifuged for 1 minute at 10,000 rpm in a bench-top centrifuge and the supernatant was collected and stored at -80°C. The amount of protein in each sample was quantified by performing a Bradford protein quantification assay. Six bovine serum albumin (BSA) standards (ranging from 0–2mg/mL) were prepared by diluting the 2mg/mL BSA standard (Roche, Mannheim, Germany) in MilliQ water. Each brain lysate was diluted 1:10 in MilliQ water, and 5μL of each sample or standard was plated in triplicate. 25μL of the DC Protein Assay Reagent (Biorad; comprising1ml of Reagent A and 20μL of Reagent S) and 200μL of DC Protein Assay Reagent B was added to each well and the plate was placed on an orbital shaker for 15 minutes before being analysed using the FLUOstar OPTIMA microplate reader (BMG Labtech, Baden-Württemberg, Germany). Standard absorbance readings were used to calculate the protein concentration for each lysate.

10μg of protein lysate was combined with 10μL of 4x Bolt LDS sample buffer and 4μl of reducing agent (500mM Dithiothreitol) and made up to 40μl with MilliQ water, before each sample was incubated at 70°C for 10 minutes. Precast polyacrylamide 4%-12% Bolt Bis-Tris Plus Gels (Life Technologioes, Carlsbad, USA) were prepared according to the manufacturers instructions. 10μL of Seablue Plus2 (Life Technologies) protein ladder was added to the first well and 40μL of lysate to the remainder. The gel was run at 140 volts for 45 minutes at 21°C, before being removed from it casing and left to equilibrate in 1x transfer buffer (5% Bolt Tranfer buffer / 10% Ethanol / MilliQ water) for 10 minutes. The gel sandwich was constructed according to the manufacturer’s instructions and the protein was transferred onto an ethanol-activated PVDF membrane (Biorad) for 60 minutes at 20 volts and 4°C. The membrane was transfered into blocking solution [5% (w/v) skim milk powder, 0.05% (v/v) tween-20 in tris buffered saline (TBS)] and incubated on the orbital shaker for 60 minutes at 21°C, before being transferred into blocking solution containing rabbit anti-LRP1 (1:40000, ab92544, Abcam) and mouse anti-GAPDH (1:40000, AB2302, Millipore). The membrane was incubated on the orbital shaker overnight at 4°C, before being washed 5 x 15 minutes in TBS /0.2% tween-20 at 21°C while agitating. The relevant horseradish peroxidase (HRP) conjugated secondary antibodies [goat anti-mouse HRP (1:20000, P044701-2, Dako) or goat anti-rabbit HRP (1:20000, P044801-2, Dako)] were diluted in TBS / 0.2% Tween20 and applied to the membrane for 1 hour on an orbital shaker at 21°C. The membrane was washed as described previously and exposed to equal volumes of Immobilon Western™ HRP Peroxidase Solution (Millipore) and Luminol Reagent (Millipore) for visualisation of the protein bands on an Image Station 4000M PRO, using Carestream software (Rochestern NY14608). Western blot band intensity was calculated by measuring integrated density and normalized to GAPDH protein expression levels.

### Tamoxifen administration

Tamoxifen (Sigma) was dissolved in corn oil (Sigma) to a concentration of 40mg/ml by sonication in a water bath for 2 hours. Adult (P57) *Pdgfrα-CreER*^*T2*^::*Rosa26-YFP* transgenic mice received 300mg/kg Tamoxifen by oral gavage, daily for four consecutive days.

### Tissue preparation

To examine LRP1 expression at defined embryonic stages, female mice were mated overnight and examined the following morning for the presence of a vaginal plug, which was denoted embryonic day 0.5 (E0.5). At the required gestational stage, pregnant mice were euthanized by CO_2_ exposure, the embryos harvested and their brains and spinal columns removed. Postnatal mice were terminally anesthetised with pentobarbitone (i.p 30mg/kg; Ilium) and perfusion fixed with 4% (w/v) paraformaldehyde (PFA; Sigma) in PBS at a rate of 9mL per minute for ~4 minutes. The brains were removed and sliced into 2mm thick coronal slices using a rodent brain matrix (Agar Scientific, Essex, UK), and the spinal cords dissected out of the spinal column. All tissue was immersion fixed in 4% PFA (w/v) in PBS for 90 minutes at 21°C, before being cryo-protected in 20% (w/v) sucrose (Sigma) in PBS overnight at 4°C. The following day, tissue was embedded in Tissue-Tek Cryomolds (Sakura, Alphen aan den Rijn, Netherlands) with Cryomatrix gel (Thermo Fisher Scientific), frozen and stored at -80°C.

### Immunohistochemistry

Embryonic brain (coronal) and spinal cord (transverse) 20μm cryosections were collected onto Superfrost Plus slides (Thermo Fisher Scientific). Postnatal mouse brain (coronal) and spinal cord (transverse) sections were cut to 30μm and collected as floating sections into PBS. Cryosections were exposed to primary antibodies diluted in blocking solution [10% (v/v) fetal calf serum and 0.1% (v/v) triton x100 in PBS] and incubated overnight at 4°C on an orbital shaker. Primary antibodies included rabbit anti-LRP1 (1:500, ab92544, Abcam), goat anti-PDGFRα (1:100, AF1062, R&D Systems), mouse anti-PSANCAM (1:500, MAB5324, Millipore), mouse anti-RC2 (1:100, MAB5740, Millipore), mouse anti-GFAP (1:2000, 556327, BD Pharmigen), guinea-pig anti-Iba1 (1:250, 234004, Synaptic Systems), mouse anti-CC1 (1:200, MABC200 Millipore), mouse anti-NeuN (1:200, MAB377, Millipore), mouse anti-parvalbumin (1:1000, MAB1572, Millipore) and rat anti-GFP (1:2000, Nacalitesque). The sections were washed 3 times for 10 minutes in PBS before the relevant secondary antibodies were applied and the sections again incubated overnight in the dark at 4°C. Secondary antibodies included Alexa488-conjugated donkey anti-goat IgG (1:1000, A-11055, Invitrogen), Alexa488-conjugated goat anti-mouse IgG (1:1000, A-11029, Invitrogen), Alexa488-conjugated goat anti-mouse IgM (1:1000, A-10684, Invitrogen), Alexa488-conjugated donkey anti-rat (1:500, O-6382), Alexa568-conjugated donkey anti-rabbit IgG (1:1000, A-10042, Invitrogen), Alexa568-conjugated donkey anti-mouse IgG (1:1000, A-10037), Alexa568-conjugated donkey anti-goat (1:1000, A-11057) and Alexa647-conjugated donkey anti-rabbit (1:1000, A-31573). Sections were washed 3 times for 10 minutes in PBS and mounted with fluorescent mounting media (Dako, Glostrup, Denmark). No primary antibody controls were also performed. PBS was substituted for tris buffered saline when detecting CC1. The nuclear marker Hoechst 33342 (1:1000, Invitrogen) was included in all secondary antibody combinations to visualise cell nuclei.

### Microscopy and Analysis

Slides were examined using an UltraView Spinning Disc Confocal microscope with Volocity Software (Perkin Elmer, Waltham, USA). Images were collected as z stacks with 2μm spacing using standard excitation and emission filters for DAPI, FITC (Alexa Fluor-488), TRITC (Alexa Fluor-568) and CY5 (Alexa Fluor-647). Images for quantification were collected using a 10x or 20x objective and stitched using Volocity software. Images to demonstrate cellular morphology were collected using a 20x or 40x objective. Images of the embryonic brain were taken at the level of the medial ganglionic eminence. Images of the adult brain were collected at approximately -1.28 Bregma. All images were analyzed with Photoshop CS6 (Adobe, San Jose, USA) or Image J (NIH, Bethesda, Maryland). Manual cell counts where performed to identify and quantify LRP1^+^ cells. For pixel intensity calculations, 40X images of the P60 mouse cortex were collected using equivalent microscopy settings. A region of interest was manually drawn around the cell soma and pixel intensity measured using Image J. Statistical comparisons were made using a t-test, Kruskall Wallis or ANOVA as specified and carried out using Prism (Graph Pad, La Jolla, USA).

## Results

### LRP1 is expressed in the developing and adult mouse brain

**Fig 1:**
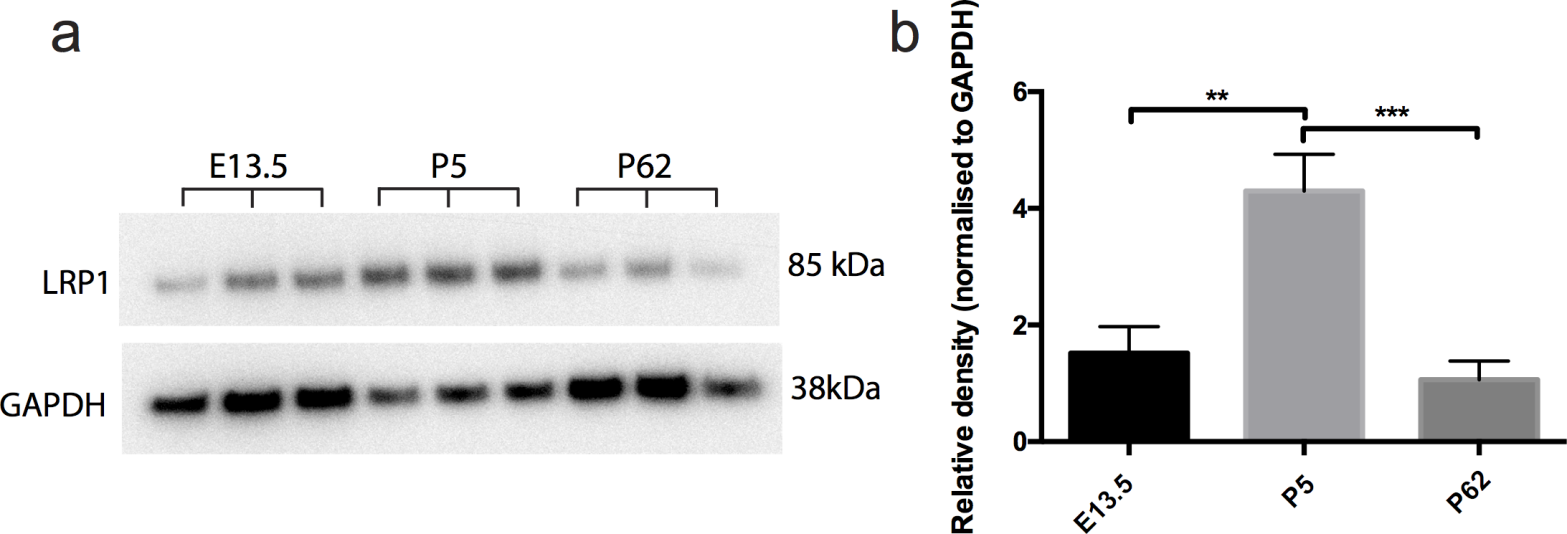
LRP1 is highly expressed in the brain. Whole brain lysates from E13.5, P5 and P62 wildtype mice were analyzed by western blot to detect LRP1 (Fig 1A) and GAPDH (Fig 1A) protein expression. (c) Band pixel intensity was quantified, normalized to the loading control (GAPDH), and shows that LRP1 expression is significantly elevated in the postnatal brain compared to the embryonic (P = 0.001) and adult (P = 0.0004) brain. Results were compared using a one-way ANOVA with a Tukey’s post-hoc test, expressed as mean ± std. **P<0.01, ***P<0.001.

The LRP1 protein is highly expressed in the brain [17]. However, the differential expression of LRP1 across development has not been investigated. To determine the relative expression of LRP1 from embryonic to postnatal development, and into adulthood, we performed a western blot analysis to detect LRP1 in protein lysates generated from E13.5, P5, and P60 C57bl6 mouse brains (n = 3 mice per age). A single 85kDa band was detected in each lysate, corresponding to the size of the beta chain of LRP1 (Fig 1A). LRP1 expression was normalised to GAPDH expression levels (Fig 1A). We found that LRP1 expression peaked during early postnatal brain development, before decreasing in adulthood (Fig 1B). From these data it is not possible to determine whether cells within the postnatal CNS reduce their expression of LRP1 with age, or whether this is the result of the changing cellular composition of the brain over this time period. To look at this more closely, and determine which cell types specifically express LRP1 in the CNS, we next undertook an immunohistochemical characterisation of LRP1 expression in the brain and spinal cord.

### LRP1 is highly expressed by radial glia in the developing CNS

**Fig 2:**
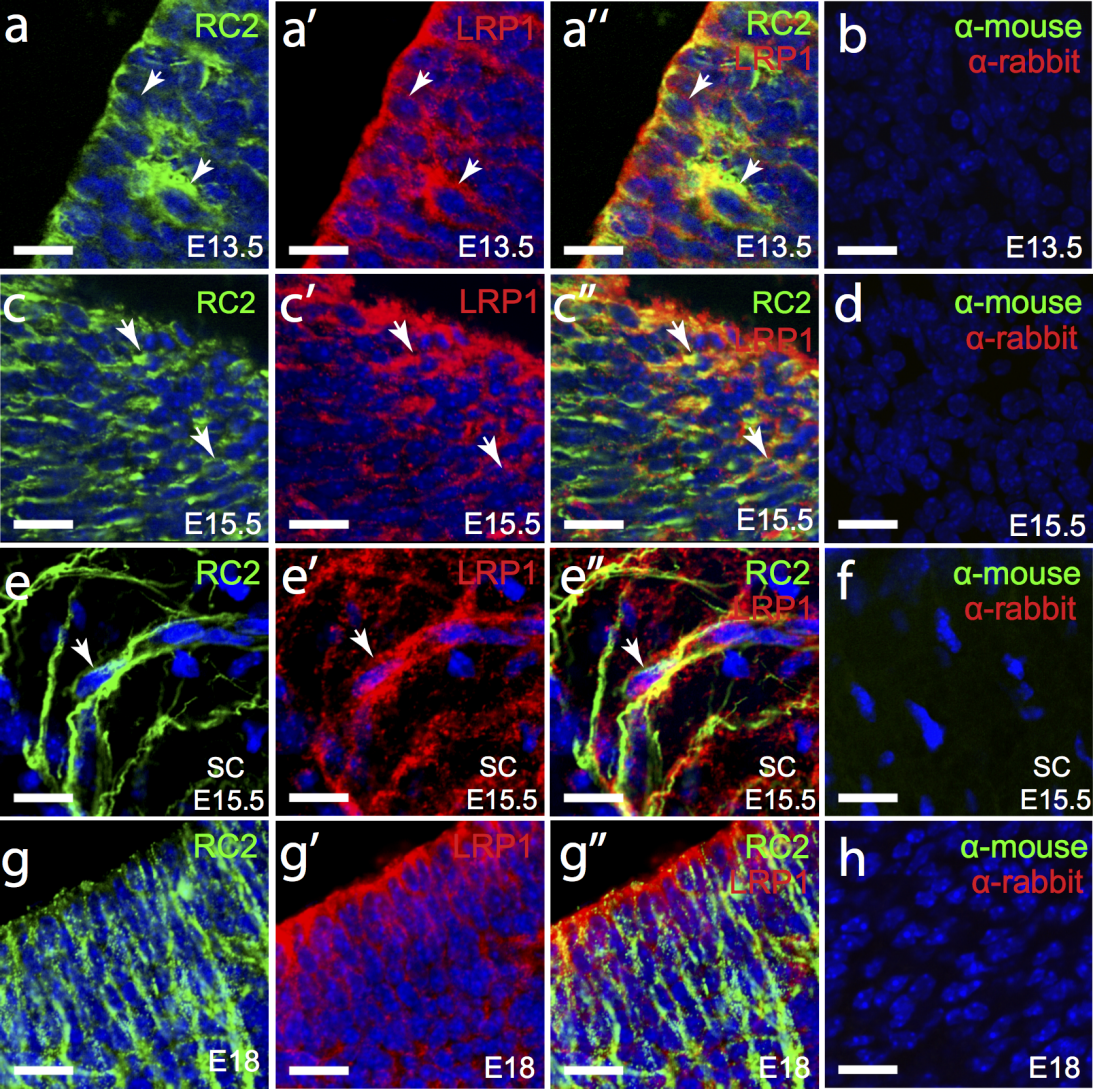
LRP1 is expressed by radial glia in the developing brain and spinal cord. Coronal sections of the E13.5 (a), E15.5 (c) and E18 (g)mouse brain and transverse sections of the E15.5 spinal cord (e) were immunolabelled to detect radial glia (RC2, green) and LRP1 (red). The nuclear marker Hoechst 33342 was used to label cell nuclei (blue). (b,d,f,h,j) secondary antibody alone controls. All images are single z plane confocal scans. White arrows indicate regions of co-localisation. Scale bars represent 17μm. SC = spinal cord.

In the embryonic brain and spinal cord, radial glial cell marker-2 (RC2) is a protein that binds to intermediate filament proteins in the radial glial stem cells [29], allowing identification of their cell bodies as well as their processes that project outwards from the neuroepithelium to the pial surface. In the E13.5 mouse brain, all radial glia (RC2^+^) were LRP1-positive (Fig 2A; 117 of 117 cells counted). Similarly, at E15.5 100% of radial glia examined in the MGE of the brain (Fig 2C; n = 125 cells counted), and in the spinal cord (Fig 2E, n = 112 cells counted) expressed LRP1 throughout the cell. At E18, LRP1^+^ cells continued to occupy the ventricular zone of the brain (Fig 2G). While it was not possible to demonstrate LRP1 co-localisation with RC2 at the cell body, due to the down-regulation of RC2, LRP1 and RC2 were still present together within the processes of these cells (Fig 2G). These data suggest that LRP1 is expressed by radial glia in the brain and spinal cord, and is sustained throughout embryonic development. Even at these early developmental stages, it was already clear that LRP1 expression was not restricted to the radial glia, as the LRP1^+^ radial glia wrapped around and made contact with other LRP1^+^ cells (Fig 2E).

### LRP1 is highly expressed by GFAP^+^ astrocytes in the postnatal CNS

**Fig 3:**
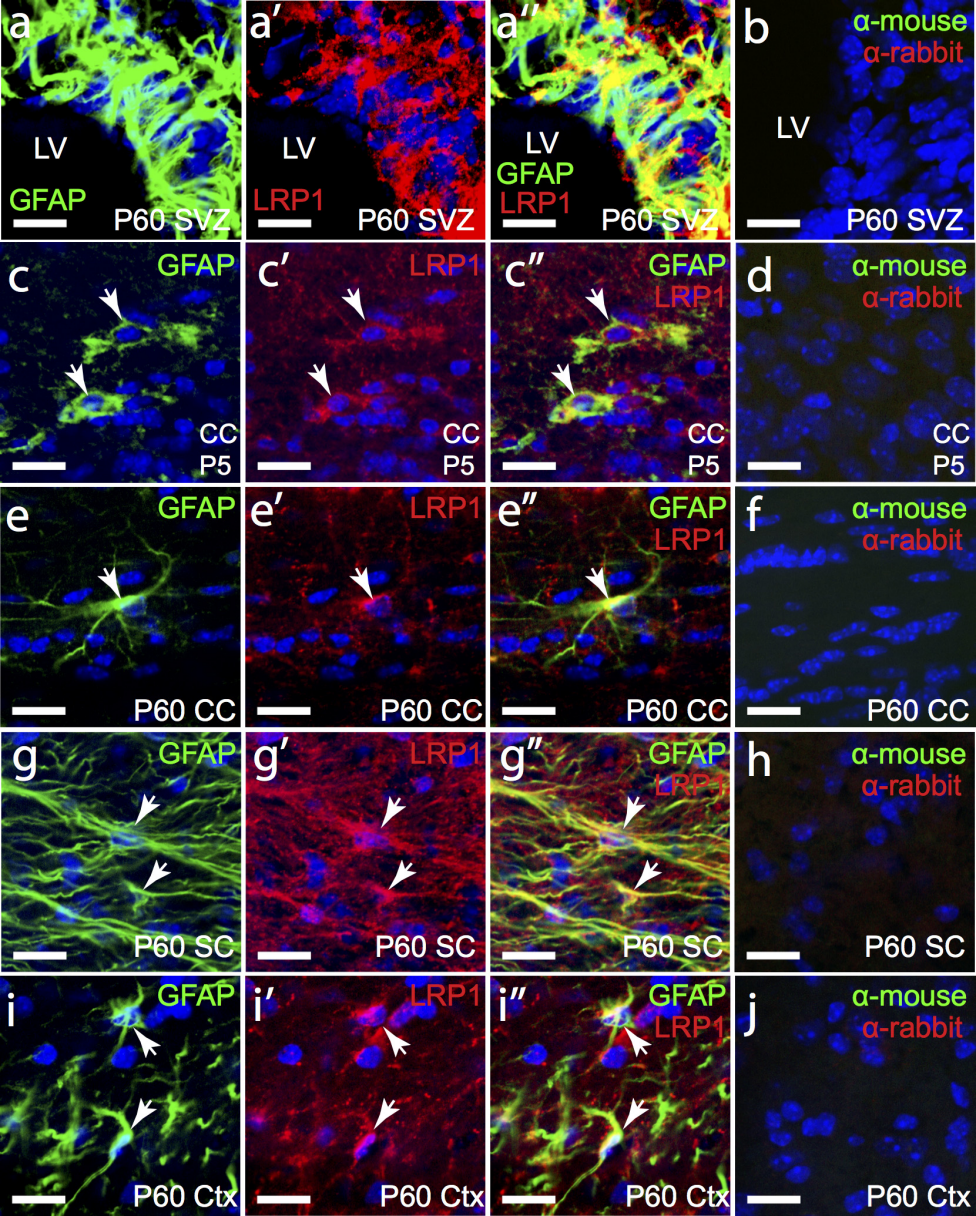
LRP1 is highly expressed by fibrous astrocytes. Coronal brain sections from P5 (c) and P60 (a,e,i) mice and transverse spinal cord sections from P60 mice (g) were immunolabelled to detect astrocytes (GFAP, green) and LRP1 (red). The nuclear marker Hoechst 33342 was used to label cell nuclei (blue). (b,d,f,h,j) secondary antibody alone controls. All images are single z plane confocal scans. White arrows indicate regions of co-localisation. Scale bars represent 17μm. CC = corpus callosum, SC = spinal cord, Ctx = cortex, SVZ = subventricular zone and LV = lateral ventricle.

Radial glia are only present during development, replaced by a population of neural stem cells in the subventricular zone (SVZ) of the lateral ventricles in the postnatal brain. These neural stem cells share a number of markers that identify them as being closely related to astrocytes. For example, fibrous astrocytes and neural stem cells both express glial fibrillary acidic protein (GFAP) [30]. GFAP^+^ cells in the SVZ of the adult brain likely comprise both of these cell populations, and were found to express LRP1 (Fig 3A; 33 of 33 cells counted). Furthermore GFAP^+^ fibrous astrocytes in the corpus callosum of the P5 mouse brain also expressed LRP1 in the soma and along their processes (Fig 3C; 73 of 73 cells counted), and this expression was retained in adulthood (Fig 3E) where 99.20% ± 1.37% of corpus callosum astrocytes expressed LRP1 (avg ± std, n = 3 mice). In the spinal cord of adult mice essentially all fibrous astrocytes were LRP1-postive (Fig 3G; 108 of 109 cells counted). In the adult mouse cortex the majority of astrocytes in the adult mouse cortex are protoplasmic astrocytes and do not express GFAP [30], however the small number of GFAP^+^ astrocytes present in layer I of the motor cortex were LRP1-positive (Fig 3I; 46 of 48 cells counted). These data are consistent with microarray [25] and RNA sequencing [26] data which indicate that *Lrp1* mRNA can be detected in astrocytes in the early postnatal mouse brain.

### LRP1 is highly expressed by neuroblasts and neurons in the developing and adult CNS

**Fig 4:**
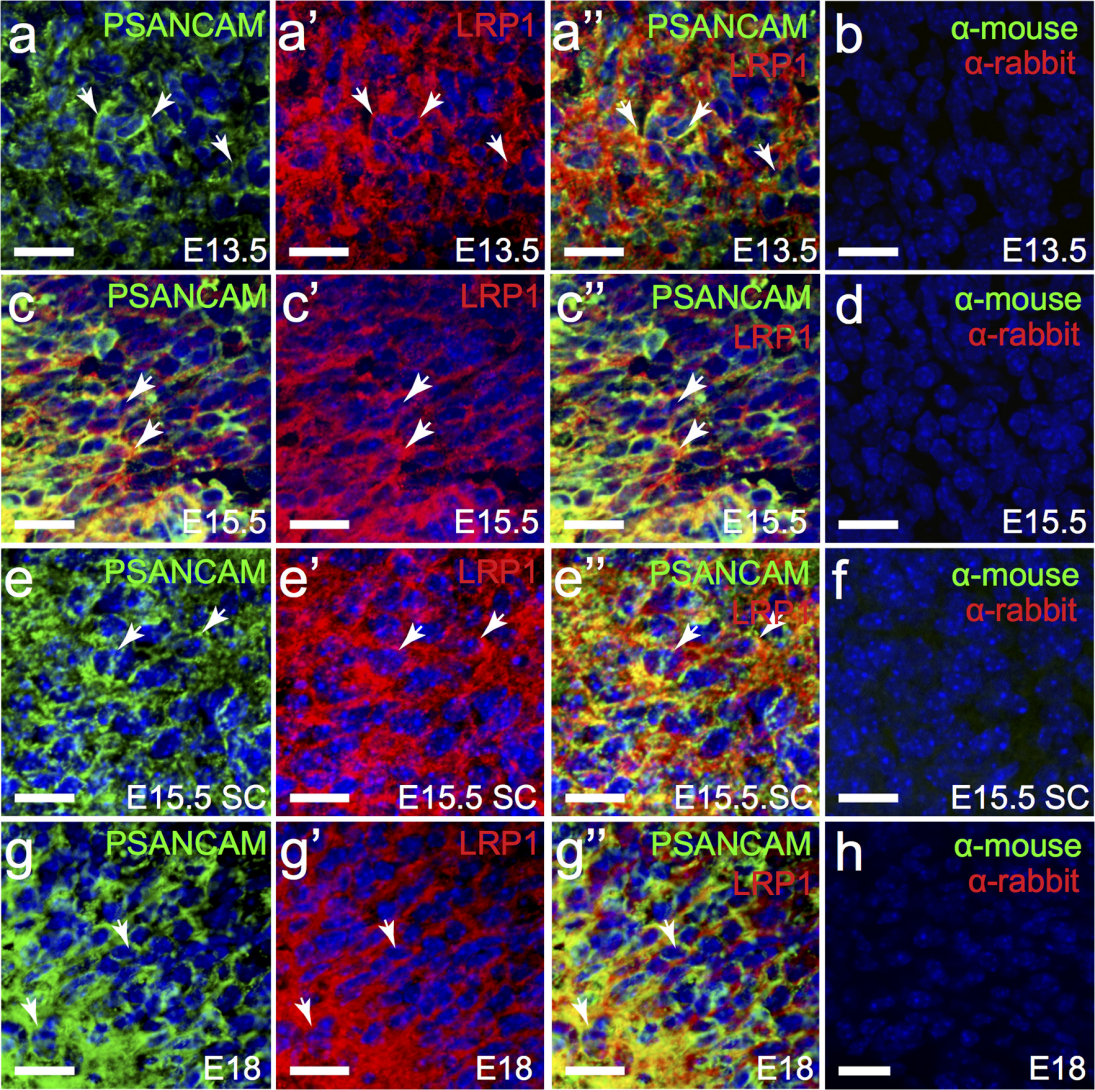
Neuroblasts in the embryonic brain and spinal cord express LRP1. Coronal sections through the embryonic mouse brain (E13.5, a; E15.5, c; and E18, g) and transverse sections of embryonic mouse spinal cord (E15.5, e) were immunolabelled to detect neuroblasts (PSANCAM, green) and LRP1 (red). The nuclear marker Hoechst 33342 was used to label cell nuclei (blue). (b,d,f,h,j) secondary antibody alone controls. All images are single z plane confocal scans. White arrows indicate regions of co-localisation. Scale bars represent 17μm. SC = spinal cord.

During development neurons are amongst the first cell type produced by radial glia. At E13.5, E15.5 and E18, PSA-NCAM^+^ neuroblasts are present throughout the telencephalon (Fig 4). This high density of neuroblasts in the MGE made quantification extremely difficult. However, at E13.5 all PSA-NCAM^+^ cells examined in the MGE of the developing brain, were found to express LRP1 (57 of 57 cells counted; Fig 4A), and continued to express LRP1 at E15.5 (108 of 110 cells counted; Fig 4C and 4E) and E18 (111 of 111 cells counted; Fig 4G).

**Fig 5:**
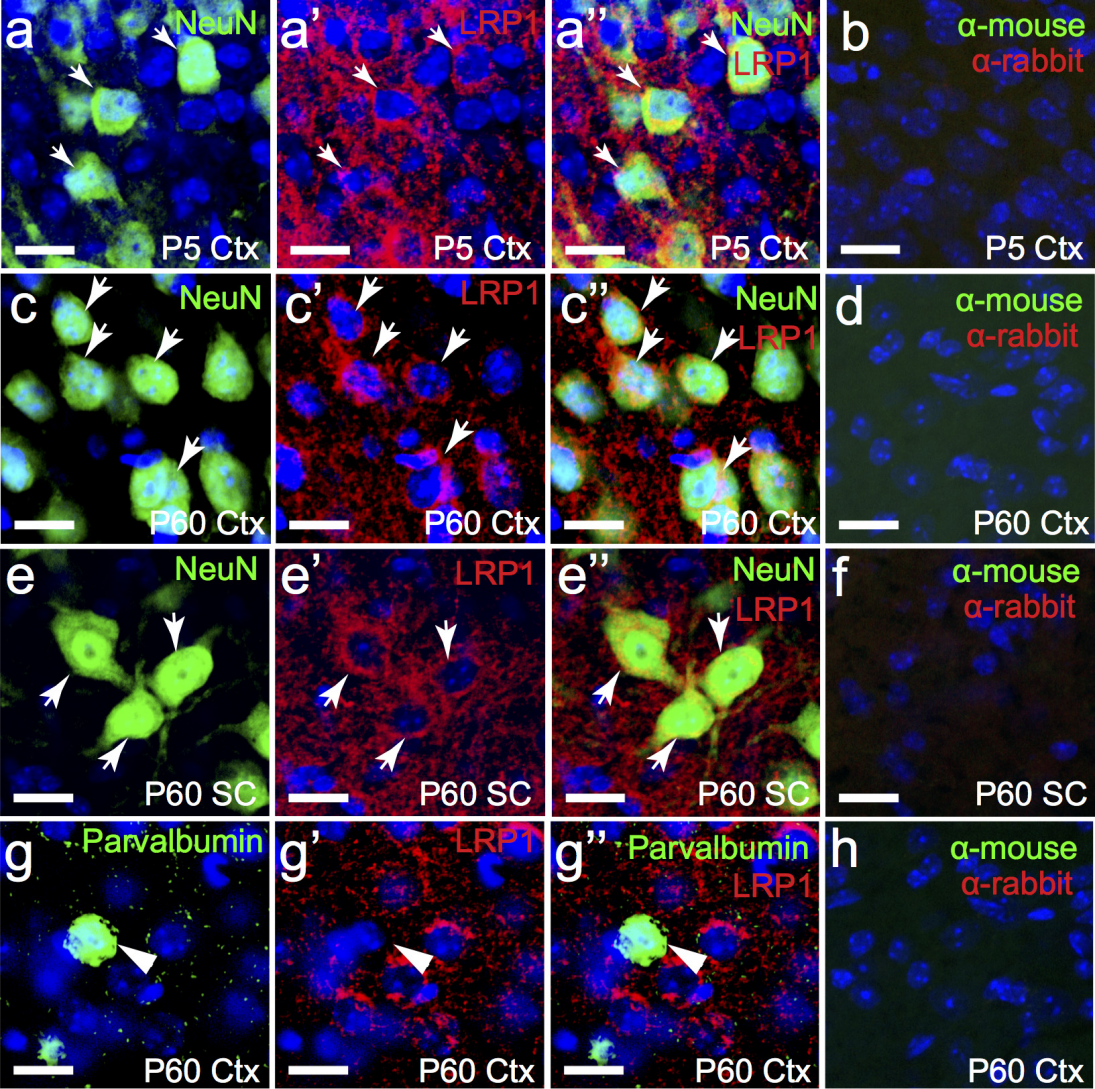
NeuN-positive neurons express LRP1, but parvalbumin-positive interneurons do not. Coronal sections through the P5 (a) and P60 (c) mouse brain, and transverse sections through the adult mouse spinal cord (e) were immunolabelled to detect mature neurons (NeuN, green) and LRP1 (red). g) Coronal section of an adult (P60) mouse brain immunolabelled to detect parvalbumin (green) and LRP1 (red). (b,d,f,h) secondary alone controls. The nuclear marker Hoechst 33342 was used to label cell nuclei (blue). All images are single z plane confocal scans. White arrows indicate regions of co-localisation. Arrow heads represent parvalbumin^+^ neurons that do not express LRP1. Scale bars represent 17μm. Ctx = cortex, SC = spinal cord.

Many of these neuroblasts mature into functional neurons in the postnatal CNS, and the fact that LRP1 is expressed by neurons is well established [11,31,32]. We determined that LRP1 was expressed by essentially all NeuN^+^ neurons in the P5 mouse cortex (111 of 113 NeuN^+^ cells counted; Fig 5A). Furthermore 98.44% ± 0.99% of NeuN^+^ cells expressed LRP1 in the adult mouse cortex (Fig 5C; n = 3 mice, avg ± std). Similarly in the spinal cord grey matter 97.7% ± 0.76% of NeuN^+^ neurons expressed LRP1 (Fig 5E; n = 3 mice, avg ± std). While NeuN is a perinuclear protein expressed by the majority of mature CNS neurons, including all excitatory neurons, many of the GABAergic inhibitory neurons of the CNS do not express NeuN [33]. However, in the cortex a large proportion of interneurons can be identified by their expression of the calcium binding protein parvalbumin. To determine whether interneurons also express LRP1, we processed P60 mouse brain cryosections to detect LRP1 and parvalbumin (Fig 5G), and were surprised to find that only 3.02% ± 2.68% of parvalbumin+ interneurons expressed LRP1 (n = 3 mice, avg ± std). These data indicate that LRP1 does not play a generic role in regulating neuron function in the CNS, and is not required for the normal functioning of parvalbumin-positive interneurons.

### LRP1 is highly expressed by microglia in the CNS

**Fig 6:**
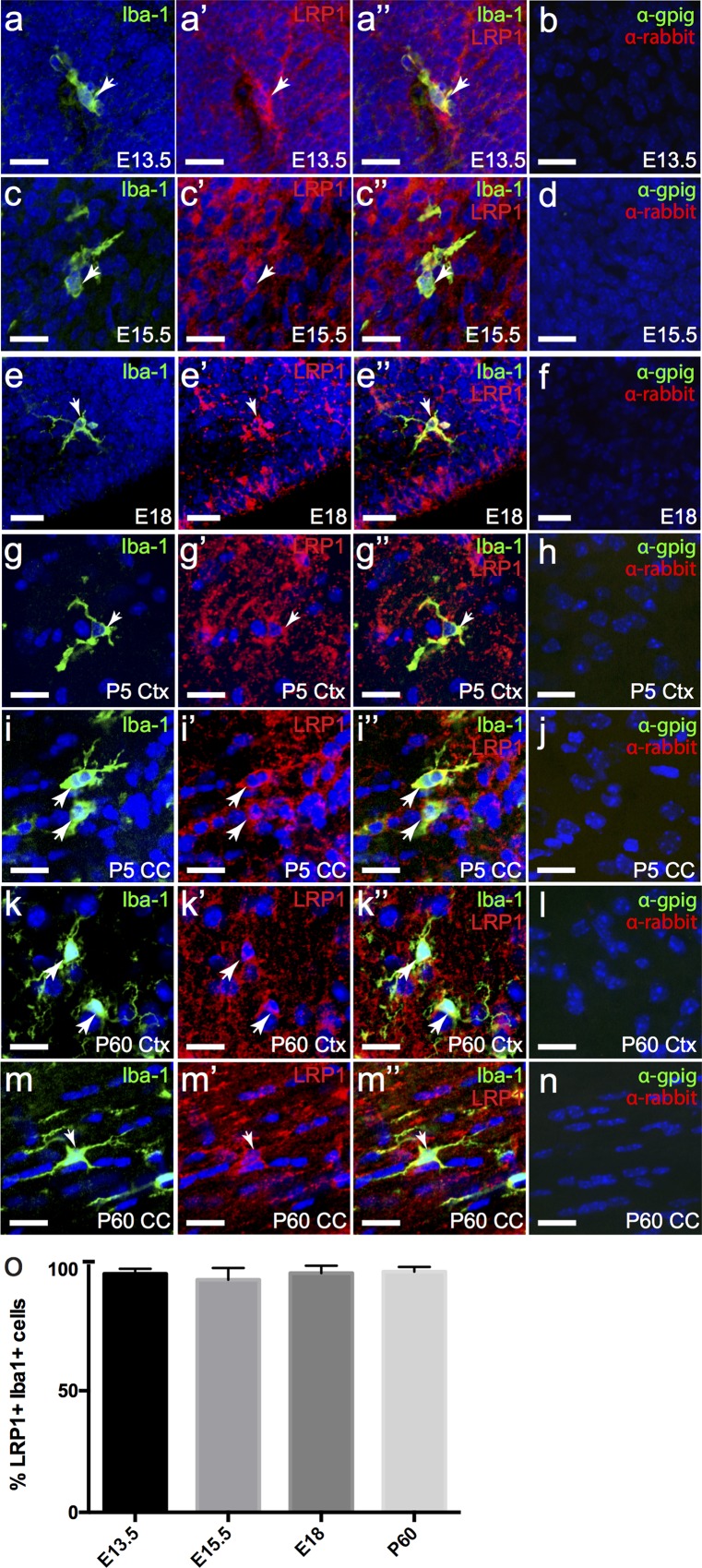
Microglia in the brain stably express LRP1 throughout life. Coronal sections of E13.5 (a), E15.5 (c), E18 (e), P5 (g, i) and P60 (k,m) mouse brain were immunolabelled to detect microglia (Iba1, green) and LRP1 (red). The nuclear marker Hoechst 33342 was used to label cell nuclei (blue). (o) Graphical depiction of the proportion of Iba1^+^ cells that expressed LRP1. Results were compared using a one-way ANOVA with a Bonferroni’s post-hoc test, expressed as means ± std and are representative of three independent experiments. (b, d, f, h, j, l, n) secondary antibody alone controls. All images are single z plane confocal scans. White arrows indicate regions of co-localisation. Scale bars represent 17μm. Ctx = cortex, CC = corpus callosum.

**Fig 7:**
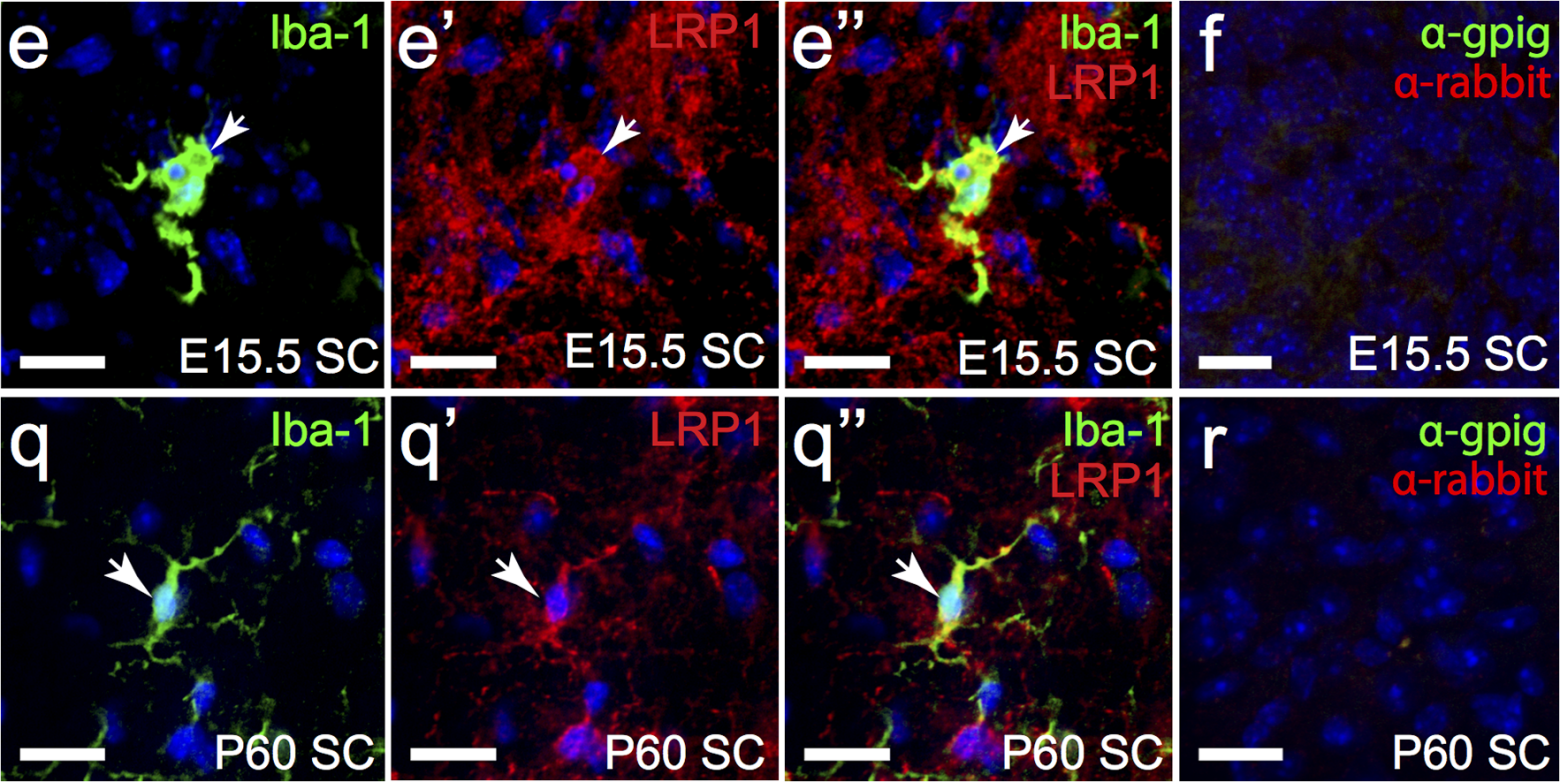
Microglia in the spinal cord express high levels of LRP1. Transverse sections of E15.5 (a) and P60 (c) spinal cord were immuno-labelled to detect microglia (Iba1, green) and LRP1 (red). The nuclear marker Hoechst 33342 was used to label cell nuclei (blue). (b,d) secondary antibody alone controls. All images are single z plane confocal scans. White arrows indicate regions of co-localisation. Scale bars represent 17μm. SC = spinal cord.

Microglia are the resident immune cells of the CNS and act as the first line of defence against CNS damage. Following CNS injury or infection, microglia alter their morphology and function to a proinflammatory, phagocytic state which allows for the clearance of cellular debris and invading pathogens [34]. *Lrp1* mRNA is reported to be highly expressed by microglia [25,26] and LRP1 has also been shown to be expressed by microglia *in vitro* [22,35,36]. To determine whether microglia express LRP1 across development and during adulthood, we performed immunohistochemistry on coronal mouse brain and transverse spinal cord cryosections to detect LRP1 (red) and the microglial marker Iba-1 (green) (Fig 6). Microglia were readily detected in the CNS at all ages, and we found that they strongly expressed LRP1 at E13.5 (Fig 6A), E15.5 (Fig 6C), E18 (Fig 6E), P5 (Fig 6G and 6I) and P60 (Fig 6K and 6M). Quantification of the proportion of microglia that express LRP1 revealed that ~96–98% of brain microglia expressed LRP1 at each age (Fig 6O). Microglia in the embryonic (Fig 7A) and postnatal (Fig 7B) spinal cord also expressed LRP1. In fact the proportion of microglia that expressed LRP1 in the brain and spinal cord was remarkably similar, with 98.66 ± 1.33% of microglia in the adult spinal cord labelling with anti-LRP1 (n = 3 mice, avg ± std). These data demonstrate that microglia consistently express LRP1 throughout development.

### LRP1 is expressed by OPCs, but not oligodendrocytes in the CNS

As their name suggests, oligodendrocyte progenitor cells (OPCs) are immature cells that give rise to the myelin-forming oligodendrocytes of the developing and adult CNS. In the mouse, the majority of oligodendrocytes are born in the first month following birth, however the life-long addition of new oligodendrocytes has been implicated in CNS repair as well as learning and memory (reviewed [37]). A recent RNA sequencing study indicated that *Lrp1* mRNA was highly expressed by OPCs, but not by oligodendrocytes [26]. However, the expression of LRP1 protein by OPCs or oligodendrocytes has never been reported.

**Fig 8:**
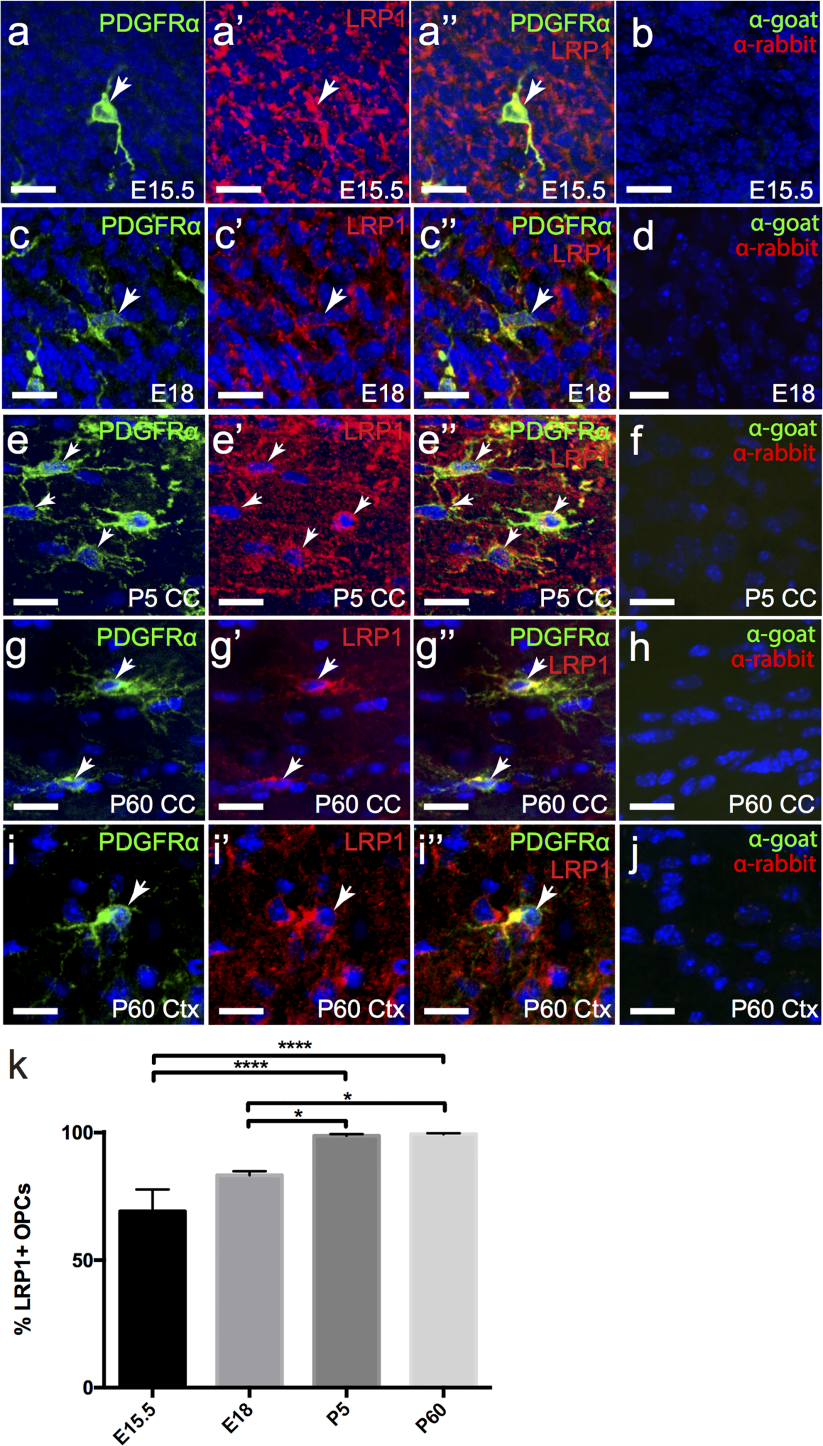
LRP1 is developmentally unregulated by OPCs. Coronal sections of E15.5 (a), E18 (c), P5 (e,i) and P60 (k,m) mouse brain were immunolabelled to detect OPCs (PDGFRα, green) and LRP1 (red). The nuclear marker Hoechst 33342 was used to label cell nuclei (blue). (b,d,f,h,j) secondary antibody alone controls. (k) Graphical representation of the percentage of OPCs that express LRP1 in the brain at each age. Data (n = 3 mice analyzed per age) were compared using a one-way ANOVA with a Bonferroni’s post-hoc test, and expressed as mean ± std. * P<0.05, **** P<0.0001. All images are single z plane confocal scans. White arrows indicate regions of co-localisation. Scale bars represent 17μm. CC = corpus callosum, Ctx = cortex.

**Fig 9:**
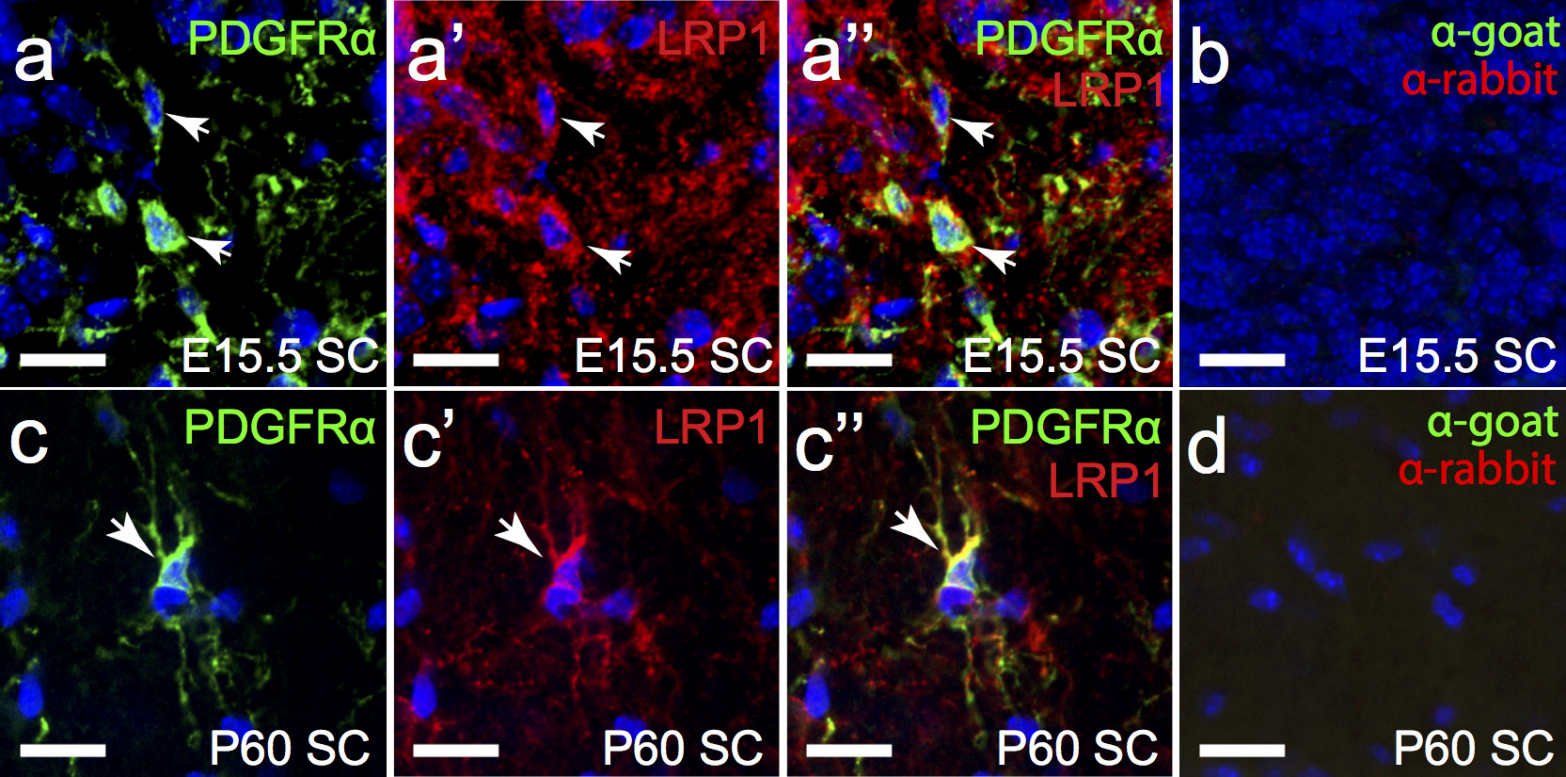
OPCs in the spinal cord express LRP1 in their cell bodies and processes. Transverse sections of E15.5 (a) and P60 (c) mouse spinal cord were immuno-labelled to detect OPCs (PDGFRα, green) and LRP1 (red). The nuclear marker Hoechst 33342 was used to label cell nuclei (blue). (b,d) secondary antibody alone controls. All images are single z plane confocal scans. White arrows indicate regions of co-localisation. Scale bars represent 17μm. SC = spinal cord.

To determine whether OPCs express LRP1 we processed cryosections to detect platelet-derived growth factor receptor α (PDGFRα; green), a protein uniquely expressed by OPCs within the CNS, and LRP1 (red) (Fig 8). By E15.5 a chain of PDGFRα^+^ OPCs extended from the MGE to the developing cortex, and ~70% of them were found to express LRP1 (Fig 8A and 8L). By E18 OPCs had populated the entire CNS [38], and the proportion that labelled with anti-LRP1 had increased to ~83% (Fig 8C and 8L), with LRP1 expression being clearly visible in the OPC soma, and throughout the processes (Fig 8C). In the P5 mouse brain ~98% of OPCs in the corpus callosum (Fig 8E and 8L) and ~99% of OPCs in the cortex (Fig 8G and 8L) labelled with anti-LRP1. Similarly, at P60, ~99% of OPCs expressed LRP1 in the corpus callosum (Fig 8I and 8L) and cortex (Fig 8J and 8L). The fraction of OPCs that expressed LRP1 was significantly less at E15.5 (p<0.0001) and E18 (p<0.05) relative to both postnatal time points examined (Fig 8L; one-way ANOVA with Bonferroni post hoc testing). The proportion of OPCs that expressed LRP1 in the embryonic and postnatal brain, was mirrored in the spinal cord, with only 77.11% ± 0.72% of OPCs expressing LRP1 at E15.5 (n = 3 mice, avg ± std; Fig 9A), but 100% ± 0% of spinal cord OPCs expressing LRP1 by adulthood (n = 3 mice, avg ± std; Fig 9C). These data suggest that OPCs acquire LRP1 expression during development, but then retain this expression throughout postnatal life.

Each OPC appeared to express a high level of LRP1 protein by immunohistochemistry. To examine this directly, we determined the maximum pixel intensity for LRP1 immunofluorescence in microglia (236 ± 8.37 arbitrary units, mean ± SEM, n = 19 cells), OPCs (208 ± 12.24 arbitrary units, mean ± SEM, n = 14 cells) and neurons (125 ± 6.68 arbitrary units, mean ± SEM, n = 14 cells) of the P60 mouse cortex. Microglia and OPCs expressed an equivalent level of LRP1, while NeuN^+^ neurons expressed significantly less LRP1 than both of these cell types (p<0.05, Kruskal-Wallis).

**Fig 10:**
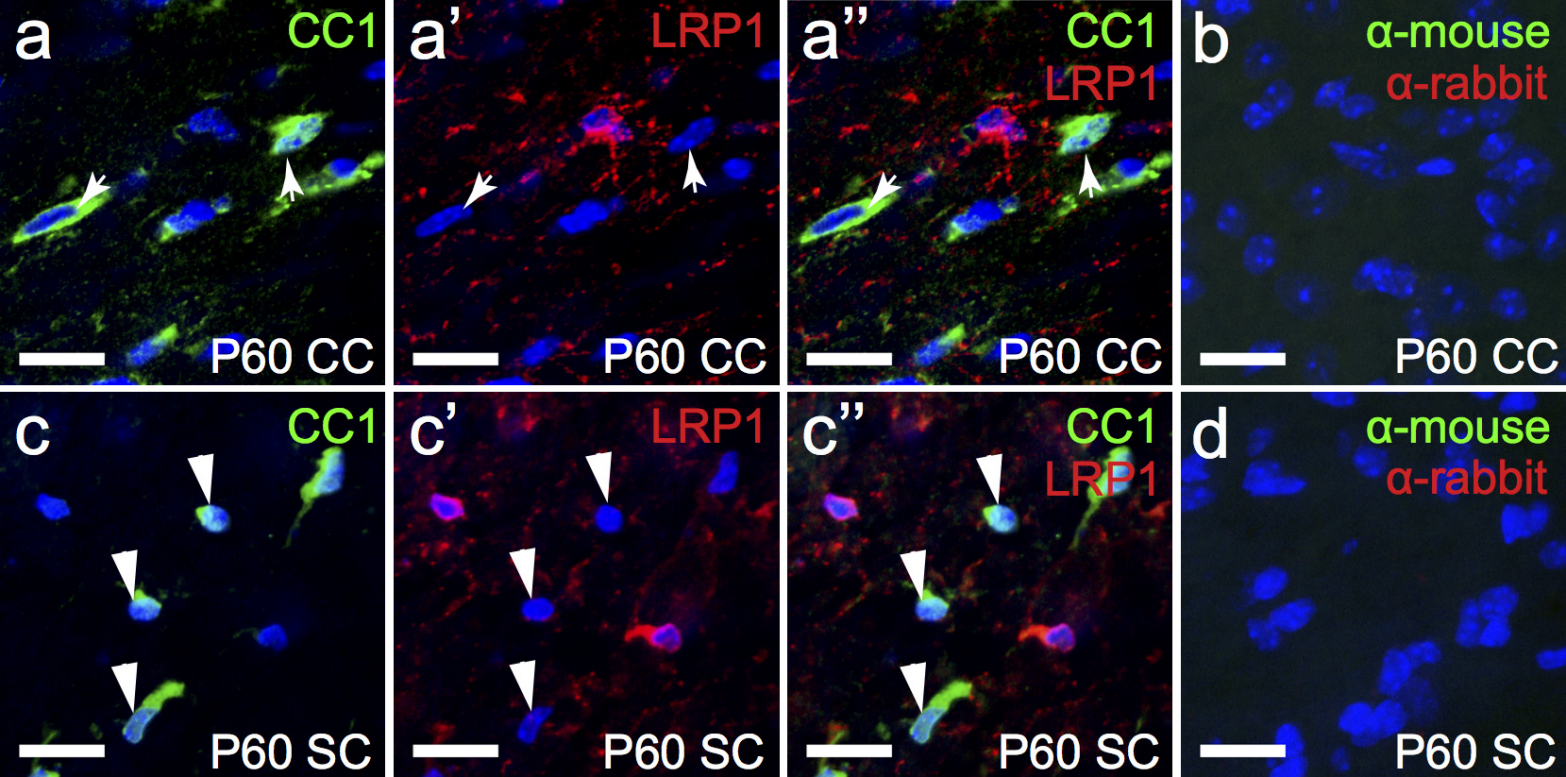
Oligodendrocytes do not express LRP1. Coronal sections through the P60 mouse brain (a) and transverse sections through P60 mouse spinal cord (c) were immuno-labelled to detect oligodendrocytes (CC1, green) and LRP1 (red). The nuclear marker Hoechst 33342 was used to label cell nuclei (blue). (b,d) secondary antibody alone controls. All images are single z plane confocal scans. Arrowheads indicate oligodendrocyte cell bodies, which do not express LRP1. Scale bars represent 17μm. CC = corpus callosum, SC = spinal cord.

When OPCs mature into oligodendrocytes they no longer express PDGFRα. Therefore, the oligodendrocyte-specific antibody CC1 was used to label oligodendrocytes in the P60 mouse corpus callosum and spinal cord white matter (Fig 10). Oligodendrocytes in the corpus callosum assemble themselves in series, running parallel with the axons that traverse the two cerebral hemispheres. We found that 0.0% ± 0.0% of CC1^+^ oligodendrocytes present in the corpus callosum were LRP1^+^ (n = 3 mice, avg ± std). Similarly, no CC1^+^ cells in the spinal cord expressed LRP1 (122 cells counted). These data indicate that oligodendrocytes do not require LRP1 for their function.

### Newly formed oligodendrocytes do not express LRP1

**Fig 11:**
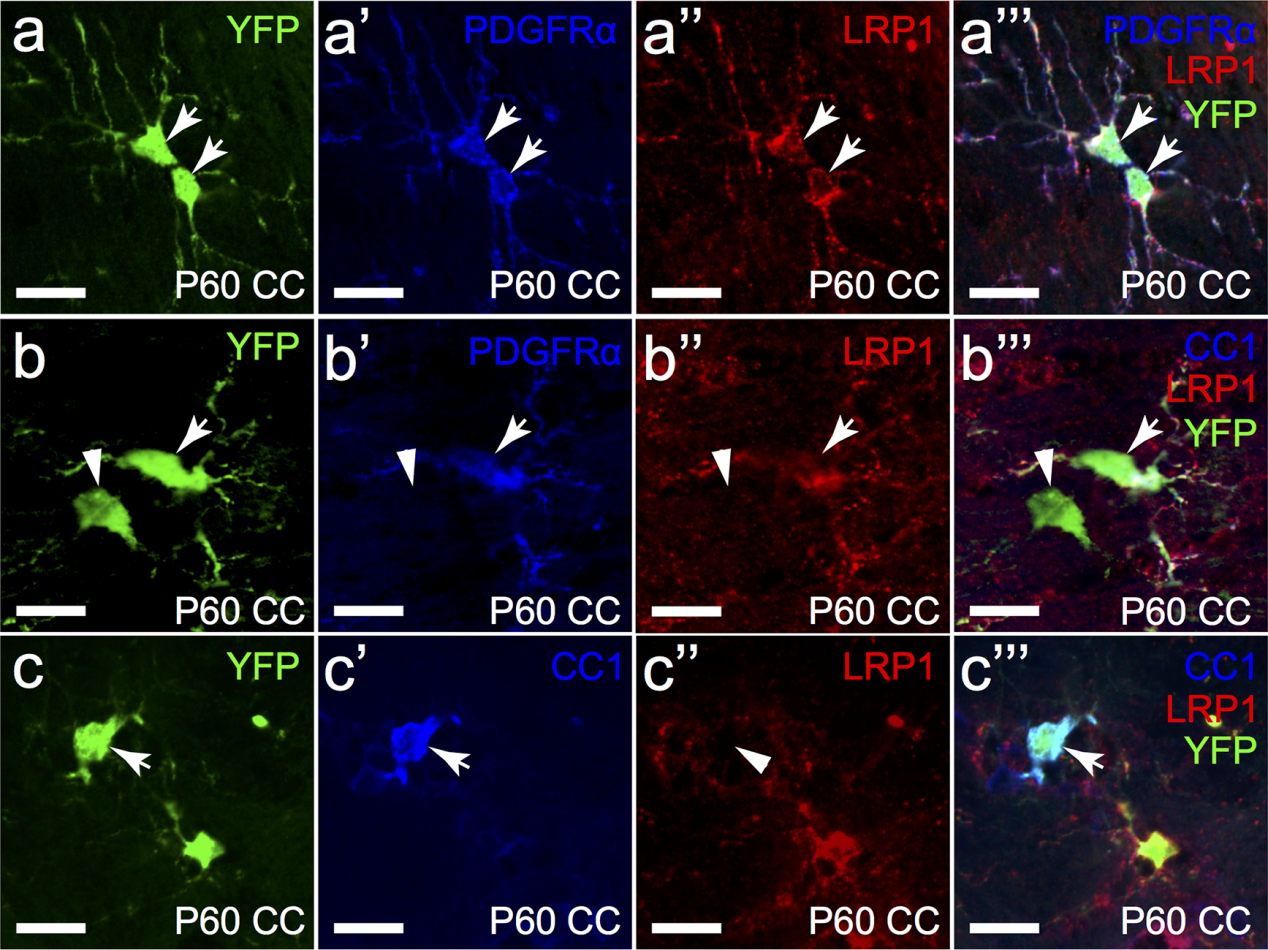
Newly formed oligodendrocytes do not express LRP1. Tamoxifen was administered to Pdgfra-CreER^T2^::Rosa26-YFP transgenic mice at P57 to label OPCs and trace them as they generate new oligodendrocytes until P64. Single scan confocal images were collected through the corpus callosum (CC) following immunolabelling with YFP (green), LRP1 (red) and either PDGFRα or CC1 (blue). **a-a”‘** YFP^+^, PDGFRα^+^ cells were also LRP1^+^. **b-b”‘** YFP^+^, PDGFRα-negative cells were also negative for LRP1. **c-c”‘** YFP^+^ CC1^+^ cells were found to be LRP1-negative. White arrows indicate regions of co-localisation. Arrowheads indicate oligodendrocyte cell bodies, which do not express LRP1. Scale bars represent 17μm. CC = corpus callosum.

Given that OPCs express a large amount of LRP1 protein, but oligodendrocytes do not, we wanted to determine when LRP1 is down-regulated. RNA sequencing data suggest that *Lrp1* mRNA is expressed by OPCs, but is still present, albeit at a lower level, in newly formed oligodendrocytes [26]. To look at this more closely we performed cre-lox transgenic lineage tracing of OPCs in adulthood. *Pdgfra-CreER*^*T2*^::*Rosa26-YFP* mice were given Tamoxifen at P57 to turn on YFP expression in PDGFRα^+^ OPCs. Mice were perfusion fixed one week later and coronal brain sections processed to detect YFP, LRP1 and either PDGFRα or CC1. As expected, we found that YFP^+^ PDGFRα^+^ OPCs in the corpus callosum had given rise to YFP^+^ PDGFRα-negative newborn oligodendrocytes in the one week tracing period [27,39]. Consistent with our earlier data, indicating that OPCs express LRP1 but oligodendrocytes do not, all YFP^+^ PDGFRα^+^ OPCs expressed LRP1 (Fig 11A, 100% ± 0%, n = 3 mice) and all YFP^+^ CC1^+^ oligodendrocytes did not express LRP1 (Fig 11B, 0% ± 0%; avg ± std, n = 3 mice). In fact, all YFP^+^ PDGFRα-negative cells were LRP1-negative (Fig 11C), indicating that LRP1 protein expression is not retained by any new born YFP-labelled oligodendrocytes, even in a population that would comprise both premyelinating and myelinating cells. These data strongly indicate that LRP1 is rapidly down-regulated alongside PDGFRα at the onset of differentiation and is not retained beyond the progenitor stage in the oligodendrocyte lineage.

## Discussion

Our data indicate that LRP1 protein is present in the brain of embryonic, early postnatal and adult mice. Specifically, LRP1 is expressed by radial glia, immature and mature neurons, excluding parvalbumin-positive interneurons, and is also expressed by microglia, astrocytes and OPCs, but not newly formed and mature oligodendrocytes. These data are largely consistent with previously published microarray and RNA sequencing studies comparing the expression of *Lrp1* mRNA by neurons, microglia, astrocytes and oligodendrocyte-lineage cells [25,26] with the exception of newly formed oligodendrocytes. The absence of LRP1 expression from oligodendrocytes may also contribute to the overall decrease in LRP1 expression detected in the brain between P5 and P60. Oligodendrocytes are largely generated after P5 in the mouse, and while this is unlikely to be the sole explanation, it would certainly be a contributing factor.

### Neuronal populations differentially express LRP1 in the mature CNS

Given that LRP1 has been implicated in neuronal development [20], it is not surprising that we observe a high level of LRP1 expression in immature neurons in the embryonic CNS. However by the time these neuroblasts mature into NeuN+ or parvalbumin^+^ neurons there is a clear divergence in LRP1 expression, as NeuN^+^ neurons expressed LRP1 while parvalbumin^+^ neurons did not. Parvalbumin-positive interneurons comprise approximately 40% of interneurons in the mature mouse cortex [40], and include interneuron subtypes such as basket and chandelier cells [41]. While this is the first study to examine the expression of LRP1 in parvalbumin-positive interneurons, a previous study reported LRP1 expression in somatostatin-positive interneurons in the hippocampus and parietal cortex [42]. Somatostatin-positive interneurons comprise approximately 30% of interneurons in the mature mouse cortex [40], and are made up predominately of Martinotti cells, as well as a small number of X94 cells [41,43]. We suspect that LRP1 may be expressed by somatostatin^+^ but not parvalbumin^+^ interneurons, due to their distinct developmental origins. Parvalbumin-positive interneurons arise from *Nkx2*.*1-*expressing precursors in the MGE, while the somatostatin-positive interneurons arise from the *Nkx6*.*2* expressing precursors in the dorsal MGE [44,45]. However this is unlikely to be the reason why parvalbumin^+^ interneurons do not express LRP1, as neuroblasts arising from the MGE at each embryonic stage examined, were LRP1-positive. Therefore, these neurons must downregulate LRP1 upon differentiation, suggesting instead that parvalbumin^+^ interneurons do not require LRP1 for their function.

We report that LRP1 is consistently expressed by NeuN-positive neurons throughout the cortex and spinal cord. These data are consistent with previous studies reporting that LRP1 expression is particularly pronounced in the cell body and proximal processes of cortical and CA1 pyramidal neurons [16–18], which are neuronal populations known to express NeuN. While this study does not examine the functional role of LRP1 in these neuronal populations, it has been previously reported that the conditional deletion of *Lrp1* from forebrain neurons *in vivo* revealed that LRP1 is important for synapse maintenance, as its absence resulted in synaptic loss and neurodegeneration. This was largely attributed to impaired lipid metabolism [32,46]. However *in vitro* studies also indicate that LRP1 interacts with post-synaptic receptors, and can thereby regulate synaptic function [19,47,48].

### LRP1 as a critical regulator of microglia in the CNS

The consistent and high level of LRP1 expression that we observed in microglia at all ages examined, points to this receptor playing an important role in regulating the behaviour of this cell type across the life-span. Previous studies have shown that LRP1 is expressed in primary cultured microglial derived from rats [49] and mice [50]. However, little is known about the role that LRP1 plays in regulating microglial function. *In vitro*, the transition of microglia from a “resting” or surveillance state to an “activated” or pro-inflammatory state can be triggered by the LRP1 ligand tissue plasminogen activator (tPA), and this same ligand can promote the migration of microglia-like BV-2 cells [36]. *In vivo*, when *Lrp1* was conditionally ablated from microglia, the cells were less responsive to cerebral ischemia [22]. However regulating the activation and migration of microglia may not be the only function of LRP1, as Lrp1 knockdown *in vitro*, reduces their phagocytic capacity, decreasing their internalisation of amyloid β [35]. These data indicate that LRP1 may be important for the initial activation of microglia, followed by migration to the site of injury and the subsequent clearing of cellular debris or foreign pathogens. Though how LRP1 differentially regulates these functions is far from understood.

### What is the function of LRP1 in astrocytes?

We have determined that LRP1 is expressed by astrocytes throughout postnatal development and into adulthood. These data are consistent with previous reports that LRP1 is expressed by human cerebral and cerebellar astrocytes [51], rat astrocytes [17] and mouse primary astrocyte cultures [49]. *Lrp1* mRNA has also been shown to be present in mouse astrocytes [26]. The role that LRP1 plays in regulating astrocytic function has not been extensively studied. However, it may be important in regulating the availability of tissue plasminogen activator (tPA) at the synapse as astrocytic LRP1 endocytoses tPA then releases it in a controlled manner [21]. Additionally, LRP1 is expressed by perivascular astrocytes, and may be involved in the regulation of blood brain barrier permeability in the early stages of cerebral ischemia [52]. Given the diverse range of functions that astrocytes perform, and the high level of LRP1 that we detect in these cells, further investigation into the function of LRP1 in this cell type would be warranted.

### What is the function of LRP1 in OPCs?

Our data raise a number of questions relating to the role played by LRP1 in regulating the behaviour of OPCs. A previous study examining cultured neurospheres found that upon differentiation, cultures that lacked *Lrp1* produced significantly fewer oligodendrocytes compared to control neurospheres [24]. The authors suggested that these data reflected a critical role for LRP1 in regulating the generation of OPCs from neural stem cells. However, an equally plausible explanation could be that LRP1 is required for the expansion of OPCs or their differentiation into oligodendrocytes.

OPCs continually produce new oligodendrocytes throughout life [27,39,53–55], and in young adult mice, the rate of oligodendrogenesis is still remarkably high [39]. By tracing the fate of OPCs *in vivo*, we were able to selectively identify oligodendrocytes that were born during a one week tracing period during adulthood. We found that the YFP-labelled newborn oligodendrocytes (PDGFRα-negative cells) were devoid of LRP1 expression. Furthermore, no CC1^+^ oligodendrocytes expressed LRP1. Our observation that newly formed oligodendrocytes did not express LRP1 was surprising due to the moderately high *Lrp1* mRNA levels identified by RNA sequencing [26], and indicate that the mRNA levels do not correlate well with protein abundance in this cell type (reviewed [56]). The rapid down-regulation of LRP1 following OPC differentiation demonstrates that LRP1 can only regulate OPC function, and is not required for oligodendrocyte maturation. Given that LRP1 expression in OPCs appears to co-localise strongly with PDGFRα (Fig 11), it is possible that these receptors form a signalling complex. There is some foundation for speculating that LRP1 may interact with PDGFRα, as it has been previously shown to interact with the related PDGFRβ in fibroblasts cell lines [57–59]. However the role of LRP1 in OPCs has not yet been investigated.

## Conclusions

LRP1 protein is present in the brain of embryonic, early postnatal and adult mice. On a cellular level, LRP1 is highly expressed by some glial and neuronal cell populations. In particular, LRP1 is expressed by radial glia, immature and mature neurons (excluding parvalbumin-positive interneurons), microglia, astrocytes and OPCs. However, LRP1 is down-regulated early in OPC differentiation, as LRP1 is not expressed by newly formed or mature oligodendrocytes. Overall, these data indicate that CNS glia are highly susceptible to regulation by LRP1 signalling, a possibility that has been largely unexplored to date.
